# Synergistic Effect of MoS_2_ and SiO_2_ Nanoparticles as Lubricant Additives for Magnesium Alloy–Steel Contacts

**DOI:** 10.3390/nano7070154

**Published:** 2017-06-23

**Authors:** Hongmei Xie, Bin Jiang, Xingyu Hu, Cheng Peng, Hongli Guo, Fusheng Pan

**Affiliations:** 1 College of Mechanical and Electrical Engineering, Yangtze Normal University, Chongqing 408100, China; xiehongmei@yznu.cn (H.X.); guohongli1211@163.com (H.G.); 2 College of Materials Science and Engineering, National Engineering Research Center for Magnesium Alloys, Chongqing University, Chongqing 400044, China; fspan@cqu.edu.cn; 3 Chongqing Academy of Science and Technology, Chongqing 401123, China; 4 College of Materials Science and Engineering, Fudan University, Shanghai 200433, China; 16307110487@fudan.edu.cn

**Keywords:** SiO_2_/MoS_2_ hybrids, lubricant additive, magnesium alloy, tribological properties

## Abstract

The tribological performances of the SiO_2_/MoS_2_ hybrids as lubricant additives were explored by a reciprocating ball-on-flat tribometer for AZ31 magnesium alloy/AISI 52100 bearing steel pairs. The results demonstrated that the introduction of SiO_2_/MoS_2_ hybrids into the base oil exhibited a significant reduction in the friction coefficient and wear volume as well as an increase in load bearing capacity, which was better than the testing results of the SiO_2_ or MoS_2_ nanolubricants. Specifically, the addition of 0.1 wt % nano-SiO_2_ mixed with 1.0 wt % nano-MoS_2_ into the base oil reduced the friction coefficient by 21.8% and the wear volume by 8.6% compared to the 1.0 wt % MoS_2_ nanolubricants. The excellent lubrication behaviors of the SiO_2_/MoS_2_ hybrid nanolubricants can be explained by the micro-cooperation of different nanoparticles with disparate morphology and lubrication mechanisms.

## 1. Introduction

Magnesium and its alloys exhibit many desirable intrinsic properties, such as low density(1.35–1.85 g/cm^3^), high specific strength and stiffness, excellent electromagnetic shielding characteristics, and abundant resources [1,2,3,4]. These favorable performances can be attributed specifically to the aspect of weight savings in design and construction for the automotive industry, mobile phones, aerospace components, and computers [5,6,7]. Especially, wrought Mg alloys, such as extruded profiles, rolled sheets, and forgings, possess better mechanical properties as compared to cast Mg alloys due to the ultrafine grain and the homogeneous distribution of the chemical composition after the plastic deformation [8]. However, the high friction and pick up of work material to the tool surfaces are inevitable issues in the cold forming process for magnesium alloys [9].

Controlling friction by lubrication is important in metal forming not only for reducing energy consumption but also for enhancing the surface quality of the formed components and the forming limitations [10]. Nonetheless, so far few lubricants are exclusively used for the forming process of Mg alloy, and in some cases, the forming lubricants used for Al alloy forming are casually used and the consequences are not favorable. Normally, sulfur, chlorine, and phosphorous containing organic compounds have been employed in the forming fluids for the Al alloy. The above mentioned organic molecules as lubricant additives play a significant role in the formation of a trio-chemical film on the contact surfaces to resist the local contact pressure. However, the poor stability of these additives during the process of applications does not meet the demands of new generation mechanical devices [11]. Additionally, the use of chlorine and phosphorus containing compounds is currently the focus of environmental concerns [12]. As a result, there is continuous research for the investigation of environmentally acceptable and efficient lubricant oil additives [13]. To date, many studies have shown that the lubrication performances for magnesium alloy/steel pairs could be significantly improved by introducing N-containing compounds [14], borates [15], and ionic liquids [16] into mineral oil. It was found that all of the compounds mentioned above as lubricant additives can interact with the surface of the magnesium alloy and generate a tribo-chemical film, thus enhancing the lubrication performance. Unfortunately, many nitrogen heterocyclic compounds showed good abrasion resistance and corrosion inhibition, but the friction reduction property was undesirable [17]. The borate without an active element, i.e. nitrogen, sulfur, and chlorine, is invalid for friction reducing and wear resistance for magnesium alloy/steel pairs. Additionally, the borates are inclined to hydrolyze, leading to the liberation of an oil-insoluble and abrasive boric acid because of the electron-deficient boron [18]. The widespread application of ionic liquids has been hindered by problems related to the thermo-oxidation, corrosion, and the cost associated with preparation [19]. Therefore, developing lubricant additives for magnesium alloys is imperative.

Recently, the prospect of lubricant additives has been expanded with the advent of nanomaterials [20]. There are many reasons to select nanoparticles as a lubricant additive. The essential feature is their nano-scale size that allows the particles to enter the contact area, and therefore the lubricant performance is improved. In addition, nanoparticles are being seen as one of the best possible options to save the environment from further pollution and degradation [21]. Different researchers have tried a variety of nanomaterial-dispersed base oil to enhance friction reduction and anti-wear behavior. Among these nano-based additives, it is reported that SiO_2_ nanoparticles are considered as a hard and brittle material easily obtained on the market in a broad range of sizes at low cost, and have been studied for the machining and drilling of Al alloy [22,23]. These results showed that an oily nano-SiO_2_ suspension was an effective lubricant providing low friction coefficient and excellent surface quality of the product. They proposed that the lubricating properties of the SiO_2_ nanoparticles were a result of an efficient rolling mechanism at the tool-chip interface. Furthermore, MoS_2_ nanoparticles are arguably the most common lubricant additive. The early studies reported that MoS_2_ nanoparticles very effectively reduce the friction and wear in the boundary-lubrication for steel-steel pairs [24,25] and titanium-steel contacts [26], and can also enhance the lubrication even on relatively inactive surfaces, such as diamond-like carbon coating/steel contacts [27,28]. The key mechanism for reducing friction and wear is associated with the weak Vander Waals interaction between layers and the formation of a tribo-chemical film on the interface. In our previous work, the tribological performances of MoS_2_ nanoparticles and SiO_2_ nanoparticles as additives in oil-based lubricants were investigated by a ball-on-flat tribometer for magnesium alloy/steel pairs and we found that MoS_2_ nanoparticles possess better anti-wear performance than SiO_2_ nanoparticles, while SiO_2_ nanoparticles obtain better dispersion in lubricating oil than MoS_2_ nanoparticles [29]. Although individual MoS_2_ nanoparticles or SiO_2_ nanoparticles showed some enhanced tribological properties, the SiO_2_/MoS_2_ hybrids as lubricant additive were expected to be more interesting.

The purpose of the present work is to determine the tribological effect of the combinative addition of MoS_2_ nanoparticles and SiO_2_ nanoparticles into the mineral oil to be used for magnesium alloy forming lubricants by a reciprocating mode of ball-on-flat [30]. The tribological performances of SiO_2_/MoS_2_ hybrid nanolubricants were evaluated in comparison with the individual nano-MoS_2_ at a concentration of 1.0 wt % in mineral oil. Furthermore, the lubrication mechanism is discussed in detail by examining the worn surfaces on the tested flats. This paper paves the way to further studies of magnesium alloys with nanolubrication.

## 2. Experimental

### 2.1. Materials

A common mineral oil (EOT5#) was selected as the base oil in the present tribo-evaluation, which was widely employed in the cold forming process of non-ferrous metal. The primary characteristics of the base oil are shown in Table 1. Based on the information provided by the supplier (Hasitai Lubricant Co., Ltd., Shanghai, China), we can exclude the existence of sulfur, phosphorus, and chlorine containing additives in the base oil. 

**Figure 1:**
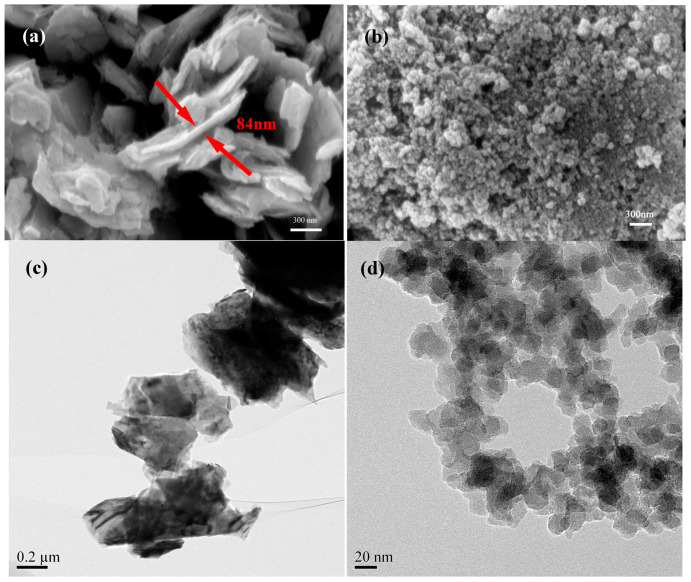

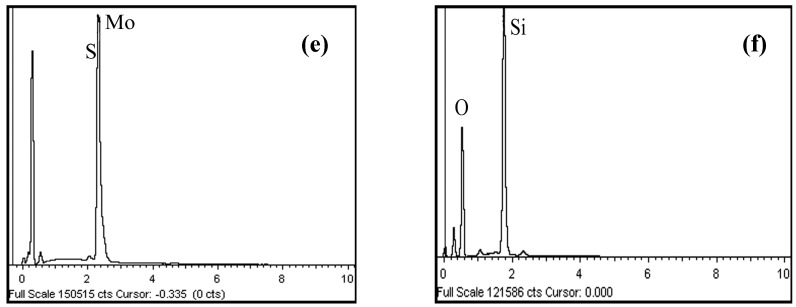
FESEM micrograph, TEM image, and EDS spectra of MoS_2_ nanoparticles (**a**,**c**,**e**) and SiO_2_ nanoparticles (**b**,**d**,**f**).

The MoS_2_ nanoparticles and SiO_2_ nanoparticles used in this study were supplied by Nanjing Emperor Nano Material Co., Ltd. (Nanjing, China). The particle size and morphology of the nanoparticles were observed with Field Emission Scanning Electron Microscope (FESEM) and Transmission Electron Microscope (TEM) (Figure 1). The results showed that the MoS_2_ nanoparticles have a flaky shape mostly with a length above 300 nm and a thickness of about 90 nm. The SiO_2_ nanoparticles have a spherical shape with a diameter of 30 nm. The optimum nano-MoS_2_ concentration (1.0 wt %) obtained by the previous screen test was adopted for the additives in the test [29]. The different concentrations of nano-SiO_2_ (0.05 wt %, 0.1 wt %, 0.2 wt %, 0.5 wt %, 0.7 wt %, 1.0 wt %) mixed with 1.0 wt % nano-MoS_2_ was added to EOT5# mineral oil by ultrasonication for 2 h to acquire a series of homogeneous nanolubricants. For comparison, 1.0 wt % MoS_2_ nanolubricants and 0.1 wt % SiO_2_ nanolubricants were prepared with the same procedure. Dispersants were not used in order to isolate the influence of the nanoparticles as lubricant additives.

### 2.2. Friction and Wear Tests

The friction reducing and anti-wear properties of the base oil and the nano-material dispersed oils were evaluated by a reciprocating mode of the ball-on-flat tribometer (CSM Instruments, Peseux, Switzerland). The upper ball used in this study is AISI 52,100 bearing steel with a hardness of 697 ± 17 kgf/mm^2^. The diameter of the ball is 6 mm and the average surface roughness of the ball is approximately 0.05 μm. The lower specimens, 10 mm × 20 mm × 3 mm in size, made of an extruded AZ31 magnesium alloy sheet (Ra = 0.08 μm, Hv = 66.7 kgf/mm^2^),were tested. Firstly, the effect of the SiO_2_/MoS_2_ mixing ratio (wt/wt) on the lubrication properties of the nanolubricants was investigated. The applied load in this study was 3 N (312 MPa of maximum Hertzian contact stress), which is at least 30% higher than the yield strength of the AZ31 magnesium alloy sheets. The sliding speed was selected as 0.08 m/s, and the duration of the test was 30 min. The optimal proportion of the SiO_2_/MoS_2_ hybrids was gained from the experiments mentioned above. Later, the effect of load on the mean friction coefficient was also studied by varying the loads (1, 3, 5, and 8 N) in a 30 min test. Finally, in order to evaluate the lubrication film stability, the normal load was increased to 8 N (446 MPa of the maximum Hertzian contact pressure), the sliding speed was decreased to 0.03 m/s, and the test time was extended to 1.5 h. All of the friction tests were conducted under ambient conditions. Prior to the frictional test, sufficient lubricant was dropped onto the ball-flat contact area. Three sets of tests at the same normal load and sliding speed were carried out to acquire each datum point to validate the repeatability and precision of the test results. Each experimental point indicates an average value of the experimental tests.

The lubrication conditions in tribological contacts contain the boundary lubrication, mixed lubrication, and hydrodynamic lubrication. The boundary lubrication is especially significant for metal forming operations. Therefore, in order to simulate the metal forming process by tribological tests, the corresponding lubrication conditions should be first determined in accordance with the *λ* ratio in Equation (1), where *h*_min_ refers to the minimum film thickness separating the surfaces. The *h*_min_ is evaluated by the Hamrock-Dowson model given in Equation (2), and *R*_q_ is the composite roughness determined by Equation (3),

(1) $$\lambda =\frac{h_{\min}}{R_{q}}$$

(2) $$h_{\min}=2.8R^{\prime }{\left(\frac{\eta u_{e}}{E^{\prime }R^{\prime }}\right)}^{0.65}{\left(\frac{W_{y}}{E^{\prime }R^{\prime 2}}\right)}^{-0.21}$$

(3) $$R_{q}=\sqrt{{R_{\mathrm{ball}}}^{2}+{R_{\mathrm{flat}}}^{2}}$$

where *W*_y_ is the applied load (3 N), *R* is the radius of the ball (6 mm), *u*_e_ is the speed (0.08 m/s), *η* is the dynamic viscosity of the nanolubricants, and *E*’ and *R*’ are the elastic modulus and effective radius for the sliding surface, respectively. The lubrication regime is generally considered by the following regulations: 0.1 < *λ* < 1 indicates boundary lubrication; 1 ≤ *λ* ≤ 3 indicates mixed lubrication, and *λ* > 3 indicates elastohydrodynamic lubrication [31]. Using the corresponding values of the fundamental constants of physics and material characteristics, the thickness of the lubricant film at the point of contact is 35 nm for 3 N at 0.08 m/s and a lambda radio of 0.37, and therefore within the boundary lubrication regime.

### 2.3. Worn Surface Analysis

After testing, the magnesium alloy flats were ultrasonically cleaned in acetone and dried in air. The wear volume on the tested flats was measured by an Olympus OLS40003D surface microscope profiler, and each scar was measured at least three times under the same conditions. The morphologies and element compositions of the worn surfaces were characterized by a Zeiss AURIGA FESEM (Zeiss; Oberkochen, Germany) equipped with an EDAX GENESIS EDS. The acceleration voltage was set at 10 kV and the working distance at 10 mm. Furthermore, the chemical species on the worn surfaces were evaluated by X-ray Photoelectron Spectroscopy (XPS, VG model Escalab 250, VG Scientific Ltd., East Grinstead, UK). The X-rays were monochromatic Al-Kα photons, and the binding energy was corrected by the adventitious C1s peak at 284.6 eV. The XPS spectra were obtained with an analyser pass energy of 29 eV, and the precision of the binding energy tests is about 0.6 eV. The measured area was approximately 500 μm in diameter. The acquired peaks were then fitted with Gaussian–Lorentzian curves after subtraction of a Shirley background using CASA XPS software (Casa software Ltd., East Grinstead, UK).

## 3. Results and Discussion

### 3.1. Friction and Wear Performances

**Figure 2:**
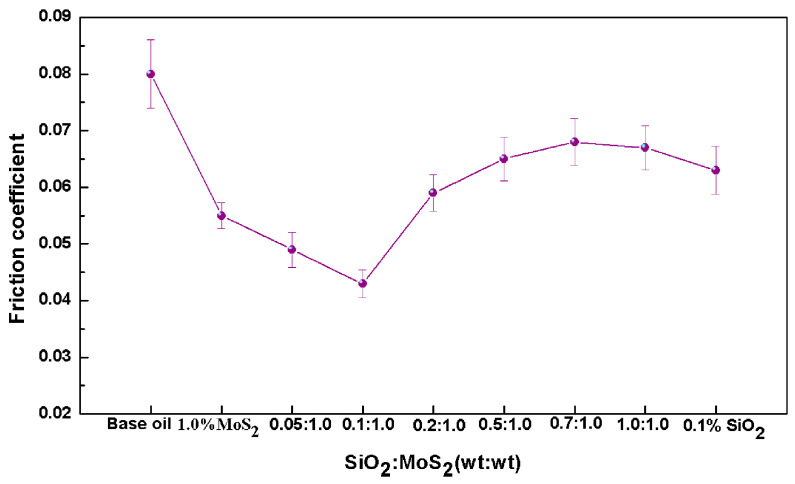
The average friction coefficient as a function of different SiO_2_/MoS_2_ nanolubricants for magnesium alloy-steel contacts (3 N, 0.08 m/s, 0.5 h).

To investigate the tribological properties of the SiO_2_/MoS_2_ hybrid nanolubricants, different mixing ratios of SiO_2_/MoS_2_ hybrid nanolubricants were prepared to test the best working conditions. Figure 2 displays the average friction coefficient of the magnesium alloy-steel pairs lubricated by various SiO_2_/MoS_2_ hybrid nanolubricants as compared with those of the individual nanolubricants at a normal load of 3 N, a sliding speed of 0.08 m/s, and the duration of 0.5 h. These nanomaterial dispersed oils show smaller friction coefficients than the base oil. This result suggests that all these nanomaterials as lubricant additives have positive effects on enhancing the friction reducing performance of the base oil. Even so, the friction-reducing efficiencies of these samples are disparate. It is observed that the friction coefficient gradually decreases with the increase of the concentration of the SiO_2_ nanoparticles in the SiO_2_/MoS_2_ hybrid nanolubricants, while a higher concentration of nano-SiO_2_ will deteriorate the friction reducing performance, which is even worse than those of the nano-MoS_2_ alone. Consequently, when the quantity of nano-SiO_2_ in the SiO_2_/MoS_2_ hybrid nanolubricants reaches an optimal concentration, a significant friction-reduction effect is obtained. For instance, when 0.1 wt % of nano-SiO_2_ is added in the SiO_2_/MoS_2_ hybrid nanolubricants, the minimum friction is acquired. The optimal concentration of nano-SiO_2_ in the SiO_2_/MoS_2_ nanolubricants reduced the friction coefficient by 21.8% compared with the 1.0 wt % MoS_2_ nanolubricants.

**Figure 3:**
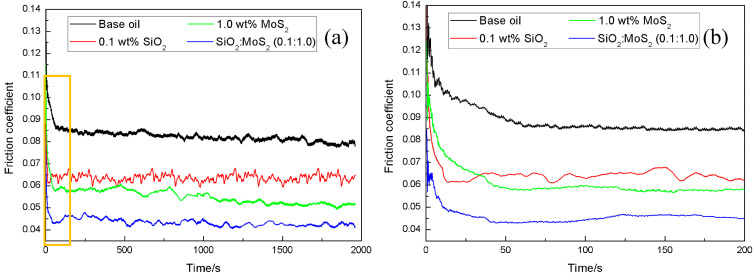
(**a**) Variation in the friction coefficient as a function of time and (**b**) the initial part of the friction coefficient curve (amplification of the yellow box) for the base oil, 1.0 wt % MoS_2_ nanolubricants, 0.1 wt % SiO_2_ nanolubricants, and SiO_2_:MoS_2_ (0.1:1.0) hybrid nanolubricants (3 N, 0.08 m/s, 0.5 h).

Figure 3 illustrates the initial part of the friction coefficient curve for magnesium alloy/steel contacts supplemented with base oil and nanolubricants. From Figure 3, it can be seen that the friction-reducing behavior of the 1.0 wt % MoS_2_ nanolubricants is slightly inferior to that of the 0.1 wt % SiO_2_ nanolubricants at the initial stage of the tests. Comparable evolution of the friction coefficient with time for steel-steel pairs lubricated by 1.0 wt % MoS_2_ nanolubricants, where a higher coefficient of friction at the initial stage of the tests can be observed, were described by L. Cizaire et al. [32]. These references indicate that the coefficient of friction is higher at the start of the tests due to the absence of the tribo-chemical film. Nonetheless, once the tribo-chemical film is generated on the contact surface during the sliding process, the friction coefficient decreased rapidly. This suggests that the tribo-chemical film provides a vital role in the friction reducing properties. The results were also verified by other studies [33]. Unlike MoS_2_ nanoparticles, SiO_2_ nanoparticles possess nano-scale size and excellent dispersion in the base oil. These properties allow the SiO_2_ nanoparticles to easily move into the contact area, thus resulting in faster running-in conditions. This is regarded as the primary reason why the friction-reducing property of 0.1 wt % SiO_2_ nanolubricants is more pronounced than that of 1.0 wt % MoS_2_ nanolubricants at the beginning stage of the tests. In sharp contrast, the friction coefficient of the SiO_2_:MoS_2_ (0.1:1.0) hybrid nanolubricants quickly decreases and stabilizes at a lower value than that of the 1.0 wt % MoS_2_ nanolubricants or 0.1 wt % SiO_2_ nanolubricants. This suggests that the synergistic effect of SiO_2_ and MoS_2_ during sliding can effectively reduce the time of the running-in stage.

**Figure 4:**
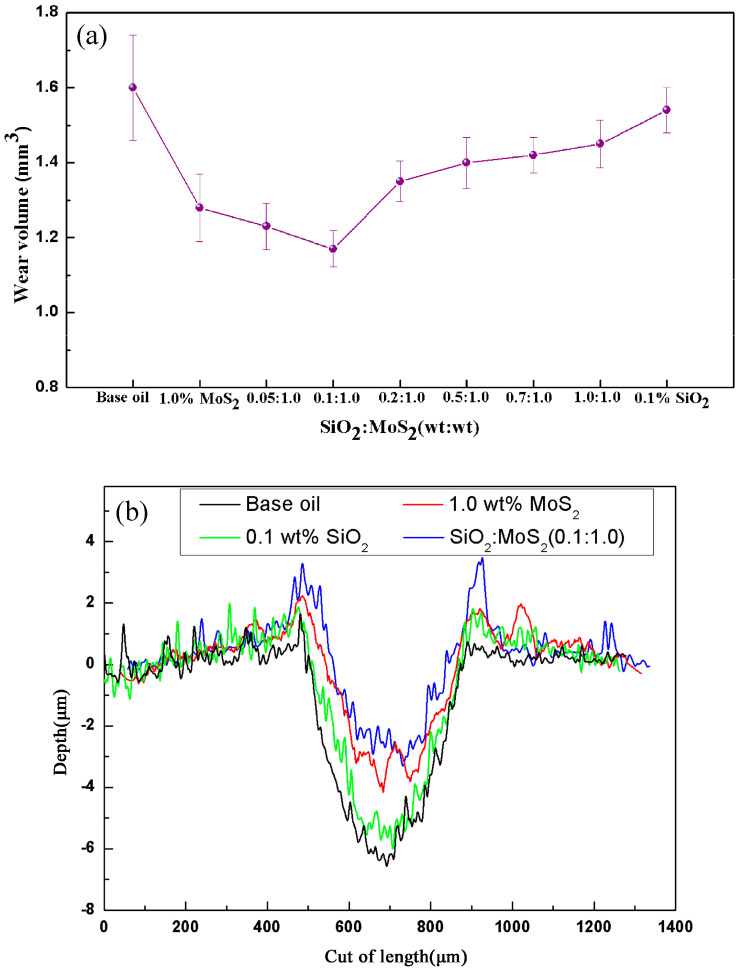
(**a**) Wear volume and (**b**) 2D cross-section profiles of the worn surface on the magnesium alloy as a function of different nanolubricants (3 N, 0.08 m/s, 0.5 h).

For the anti-wear performance, since the AISI 52100 steel ball in this study is harder than the AZ31 magnesium alloy flat, most of the wear tracks are not obvious and deep enough to accurately calculate the wear volume of the ball. Therefore, comparisons of the average wear volume and the 2D cross-section profiles of the worn surface on the corresponding AZ31 magnesium alloy lubricated by different samples are shown in Figure 4. As shown in Figure 4a, it was found that the anti-wear performance of the SiO_2_/MoS_2_ hybrid additive is better than that of the single nano-SiO_2_ or nano-MoS_2_. When an optimized concentration of nano-SiO_2_ in the SiO_2_/MoS_2_ hybrid nanolubricants reaches 0.1 wt %, the decrease of the wear volume is about 8.6% as compared with the 1.0 wt % MoS_2_ nanolubricants. Similar to the friction reducing effect, further increasing the concentration of the nano-SiO_2_ will deteriorate the anti-wear performance. As can be observed from the 2D cross-section profiles of the wear tracks lubricated by different lubrications in Figure 4b, the SiO_2_:MoS_2_ (0.1:1.0) hybrid additive exhibited shallower wear tracks than individual SiO_2_ or MoS_2_ did. In this case, the appropriate mass ratio of nano-SiO_2_ and nano-MoS_2_ appeared at 0.1:1.0. Therefore, SiO_2_:MoS_2_ (0.1:1.0) hybrid nanolubricants were selected for further study.

### 3.2. The Capacity of Carrying Load

**Figure 5:**
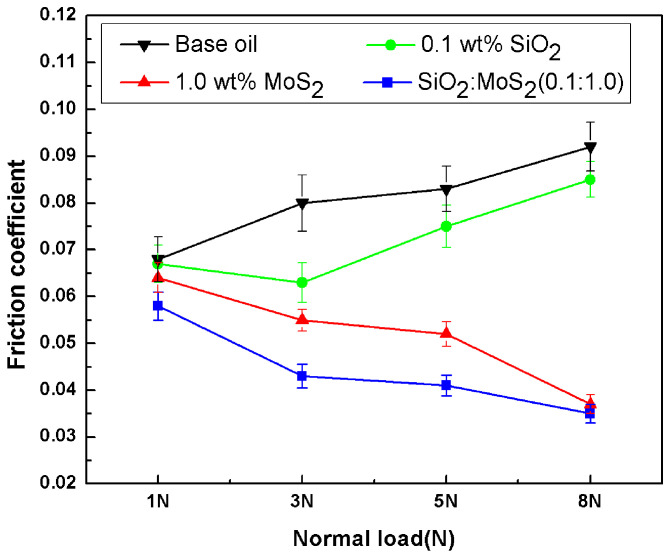
Friction coefficients for the base oil and nanolubricants under four different loads (0.08 m/s, 0.5 h).

Figure 5 depicts the average friction coefficient as a function of the load for the base oil, 0.1 wt % SiO_2_ nanolubricants, 1.0 wt % MoS_2_ nanolubricants, and SiO_2_:MoS_2_ (0.1:1.0) hybrid nanolubricants (speed, 0.08 m/s, time, 30 min,). It is observed that the friction coefficient increases with the increase of normal load for the base oil and 0.1 wt % SiO_2_ nanolubricants. This is most probably because the micro-intervals between the friction pairs decrease with an increase of the normal load. Therefore, there is less base oil that could enter into the frictional interface, resulting in an increase in the direct contact of the surface asperities. In the case of the 0.1 wt % SiO_2_ nanolubricants, spherical SiO_2_ nanoparticles may act as nano-bearings between the moving parts, thus improving the friction reduction properties. However, we need to mention that the roller bearing effect of the spherical nanoparticles during sliding is very deeply inclined with the operating conditions. The references [34,35] reported that the shape of the nanoparticles is obscured if the lubricant film thickness between the two surfaces gets close to the size of the spherical nanoparticles. In this study, the minimum film thickness (*h*_min_) calculated according to the Hamrock-Dowson equation is 44 nm, 35 nm, 31 nm, and 28 nm for 1 N, 3 N, 5 N, and 8 N, respectively. It is indicated that the lubricant film thickness between the contact surfaces decreases with increasing load, which is getting close to the size of the spherical SiO_2_ nanoparticles. Consequently, the shape of the nanoparticles is obscured during rubbing, resulting in an increase in the friction coefficient. That may be why the friction coefficient for 0.1 wt % SiO_2_ nanolubricants increased with increasing the applied load, although it still remains lower than that of the base oil. The friction behaviors of the 1.0 wt %MoS_2_ nanolubricants and the SiO_2_:MoS_2_ (0.1:1.0) hybrid nanolubricants are found to be quite different from the base oil and the 0.1 wt % SiO_2_ nanolubricants at the same experimental conditions. The friction coefficient for the 1.0 wt % MoS_2_ nanolubricants and SiO_2_:MoS_2_ (0.1:1.0) hybrid nanolubricants decreases gradually with increasing load. These results reflect the fact that the friction-reducing property of the 1.0 wt % MoS_2_ nanolubricants or SiO_2_:MoS_2_ (0.1:1.0) hybrid nanolubricants gradually strengthens with increasing load. With the increasing load, the MoS_2_ nanoparticles easily interact with the new-exposed contact surfaces. This is conducive to the formation of the tribo-chemical film on the surface, thereby decreasing the friction coefficient. Additionally, from an atomistic and computational aspect [36,37,38], the repulsive contribution of the Lennard-Jones potential increases with an increase in the applied load, thus offering the “ground” for better sliding and a lower friction coefficient. 

### 3.3. Lubrication-Film Stability

**Figure 6:**
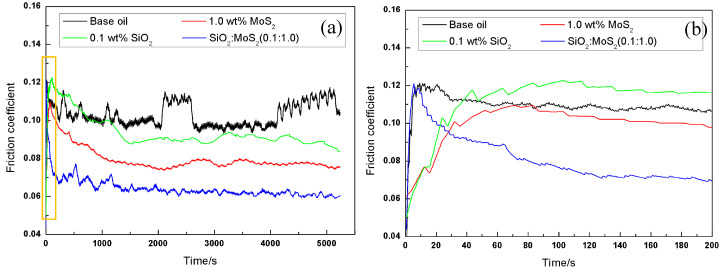
(**a**) Sliding time to lubrication-film break down and (**b**) the initial part of the friction coefficient curve (amplification of the yellow box) for the base oil, 1.0 wt % MoS_2_ nanolubricants,0.1 wt % SiO_2_ nanolubricants, and SiO_2_:MoS_2_ (0.1:1.0) hybrid nanolubricants (8 N, 0.03 m/s, 1.5 h).

In order to investigate the stability of the lubrication-film during rubbing in more extreme test conditions, the tribological experiments were conducted at a load of 8 N and sliding speed of 0.03 m/s for 1.5 h. Variations of friction coefficients of the base oil, 0.1 wt % SiO_2_ nanolubricants, 1.0 wt % MoS_2_ nanolubricants, and the SiO_2_:MoS_2_ (0.1:1.0) hybrid nanolubricants with increasing sliding time are shown in Figure 6. As shown in Figure 6a, a general order of friction coefficient values is observed as follows: SiO_2_:MoS_2_ (0.1:1.0) hybrid nanolubricants < 1.0 wt % MoS_2_ nanolubricants < 0.1 wt % SiO_2_ nanolubricants< base oil. The friction coefficient for the base oil presented acute fluctuations after about 230 s of sliding. The high friction coefficient with dramatic fluctuations is a classic indication of lubrication failure resulting in the contact surface scuffing and the propagation of scuffing damage [39]. When 0.1 wt % SiO_2_ nanoparticles were dispersed into the base oil, the friction coefficient had little difference to the base oil at the early stage of the test, as they were all about 0.11.As the test duration increased, the friction coefficient became lower and less noisy. During the sliding, nano-SiO_2_ was possibly adsorbed onto the contact areas to present the rolling effect like miniature bearings, and thus the friction coefficient decreased gradually [40]. The third curve is the 1.0 wt % MoS_2_ nanolubricants.The presence of nano-MoS_2_ reduced the friction coefficient from 0.11 to about 0.077 in the first 20 min, after which the friction coefficient approached a steady state and lasted for the entire tribological test. Interestingly, the friction-reducing behavior of 1.0 wt % MoS_2_ nanolubricants or 0.1 wt % SiO_2_ nanolubricants has little difference at the early stage of the tests (Figure 6b). Compared with the tribological tests in Figure 3, it is more difficult for MoS_2_ or SiO_2_ nanoparticles to enter the contact area at the early stage due to the higher load in the lubrication-film stability tests. Therefore, it can be inferred that the nanoparticle additives in the base lubricant have little impact on the friction coefficient at the initial stage of the tests. In contrast, the friction coefficient of the SiO_2_:MoS_2_ (0.1:1.0) hybrid nanolubricants was quite different from the other samples. The beginning stage of the friction coefficient dramatically reduced, and gradually stabilized in the latter part of the curve. The results illustrated that the SiO_2_ nanoparticles can achieve a synergic function on the friction reducing abilities of nano-MoS_2_ even under harsh tribo-conditions. 

### 3.4. FESEM and XPS Analyses of the Worn Surfaces

**Figure 7:**
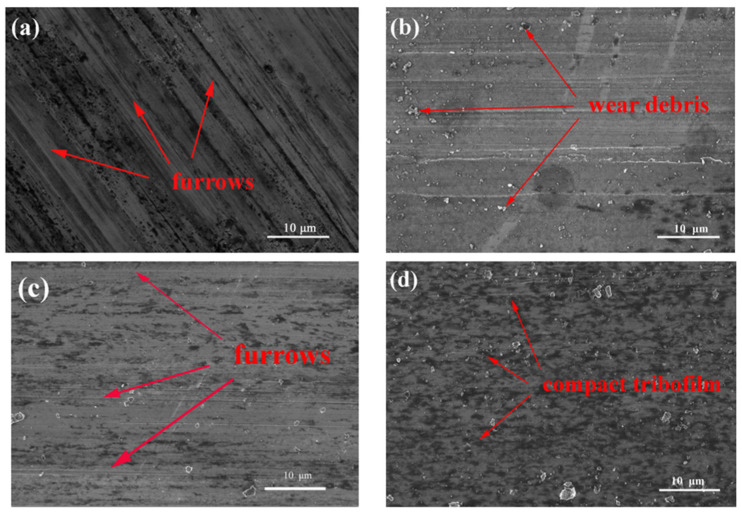
FESEM images of the worn surfaces on the magnesium alloy lubricated by: (**a**) base oil, (**b**) 0.1 wt % SiO_2_ nanolubricants, (**c**) 1.0 wt % MoS_2_ nanolubricants, and (**d**) SiO_2_:MoS_2_ (0.1:1.0) hybrid nanolubricants (3 N, 0.08 m/s, 0.5 h).

To further evaluate the effect of different lubricants on the tribological mechanism, Figure 7 shows the FESEM images of the worn surface on the AZ31 magnesium alloy after sliding for 30 min at 3 N and 0.08 m/s. As expected, the wear scar lubricated by the base oil is very rough. Numerous ridges and grooves on the entire wear scar can be found, which indicates that the base oil is not efficient at protecting the rubbing surface. For the 0.1 wt % SiO_2_ nanolubricants, the worn surface shows apparent scuffing accompanied with wear debris. The hard nano-SiO_2_ plows the soft magnesium alloy surfaces under the applied load, which resulted in the abrasive wear. Even so, the severe scuffing damages for the SiO_2_ nanolubricants are greatly reduced when compared to the base oil. It is indicated that SiO_2_ nanoparticles as a lubricant additive improves the anti-wear behavior of the base oil, while when the base oil is formulated with 1.0 wt % nano-MoS_2_, the wear process further slows down. The furrows on this wear surface become flat and smooth and the furrow numbers are also decreased. Some scattered black remnants are observed in Figure 7c, indicating that a tribo-chemical film may be generated during the rubbing process. The transfer film improved the anti-wear properties by protecting the direct contact between the magnesium alloy-steel pairs. In marked contrast, the SiO_2_:MoS_2_ (0.1:1.0) hybrid nanolubricants produce even less surface damage with wear debris and fewest grooves in a more random pattern. More importantly, a more compact tribofilm is formed on the worn surface (Figure 7d). It again proves that SiO_2_/MoS_2_ hybrids as lubricant additives can dramatically reduce the friction and wear for magnesium alloys/steel contacts. The FESEM images of the worn surface are consistent with the measured tribological characteristics.

**Figure 8:**
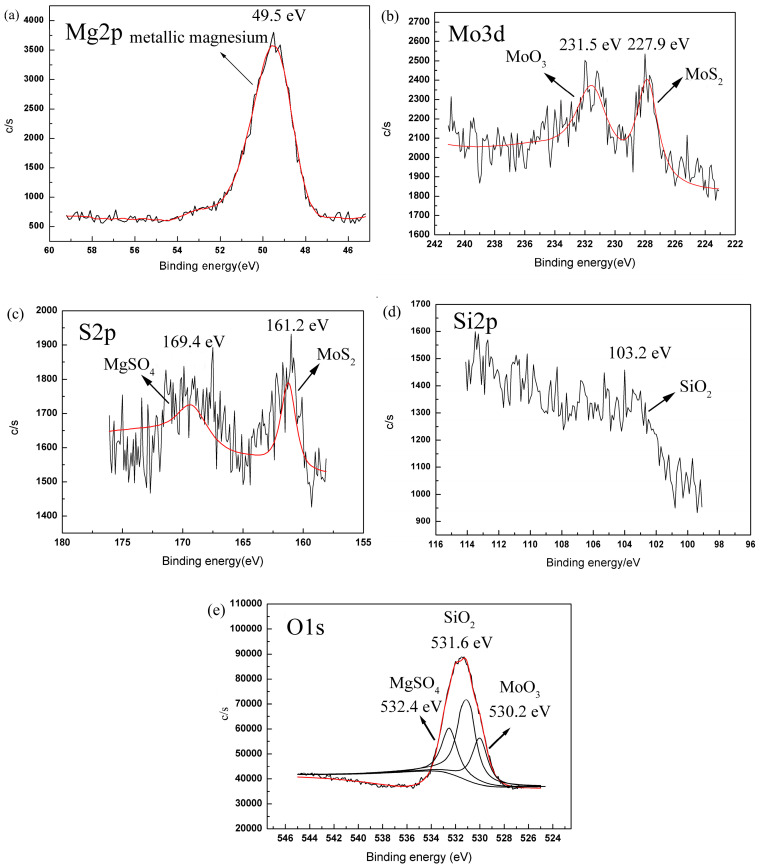
XPS spectra of (**a**) Mg2p, (**b**) Mo3d, (**c**) S2p, (**d**) Si2p, (**e**) O1s on the wear scars lubricated with SiO_2_:MoS_2_ (0.1:1.0) hybrid nanolubricants. (3 N, 0.08 m/s, 0.5 h).

To investigate the chemical composition of the tribofilm, the XPS spectra of the worn surface on the magnesium alloy lubricated by SiO_2_:MoS_2_ (0.1:1.0) hybrid nanolubricants has been recorded. As shown in Figure 8, the spectrum of Mg2p (Figure 8a) shows a peak at the binding energy of 49.5 eV, which was attributed to the chemical state of the metallic magnesium. The Mo3d (Figure 8b) peaks appeared at 228.3 eV and 231.5 eV. The former combined with the S2p peaks at 161.2 eV is assigned to MoS_2_ [41]. The latter combined with the O1s peak at 530.2 eV is assigned to MoO_3_ [42]. The MoS_2_ signal indicates that nano-MoS_2_ has been deposited on the contact surfaces and forms a physical protecting film on the flat. Furthermore, the MoO_3_ signal suggests that a few MoS_2_ nanoparticles deposited on the contact surfaces have been oxidized during the rubbing process. The S2p peak appears at approximately 169.4 eV, combined with the binding energy of O1s 532.4 eV, indicating the generation of MgSO_4_ [20]. The amount of MgSO_4_ is not enough to affect the XPS spectrum of the main element Mg in Figure 8a. The MgSO_4_ signal also indicates that nano-MoS_2_ has reacted with the magnesium alloy surface during the sliding process, and thus a new chemical transfer film formed on the worn surface. Furthermore, the Si2p spectrum (Figure 8d), associated with the O1s peak at 531.6 eV, shows a dominant peak at 103.2 eV assigned to SiO_2_ [43].It is deduced that the SiO_2_ nanoparticles cooperated with the MoS_2_ nanoparticles to lubricate the surface of the magnesium alloy. Therefore, it can be reasonably concluded that the SiO_2_/MoS_2_ hybrid nanolubricants have proven their remarkable friction reducing and anti-wear behaviors through generating a complicated boundary lubrication film that mainly consists of MoO_3_ and MgSO_4_ via chemical reaction plus the absorbed nanomaterial which comes from the lubricant additive itself.

### 3.5. Lubrication Mechanism of SiO_2_/MoS_2_ Hybrid Nanolubricants

**Figure 9:**
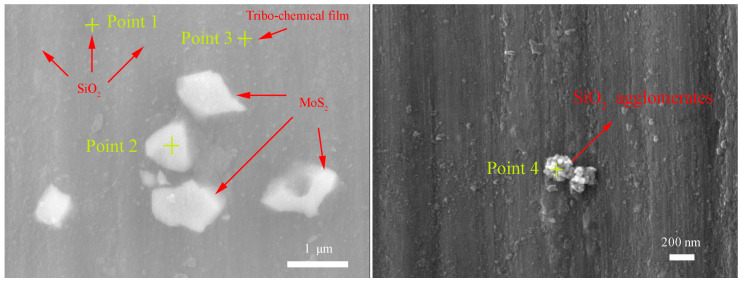

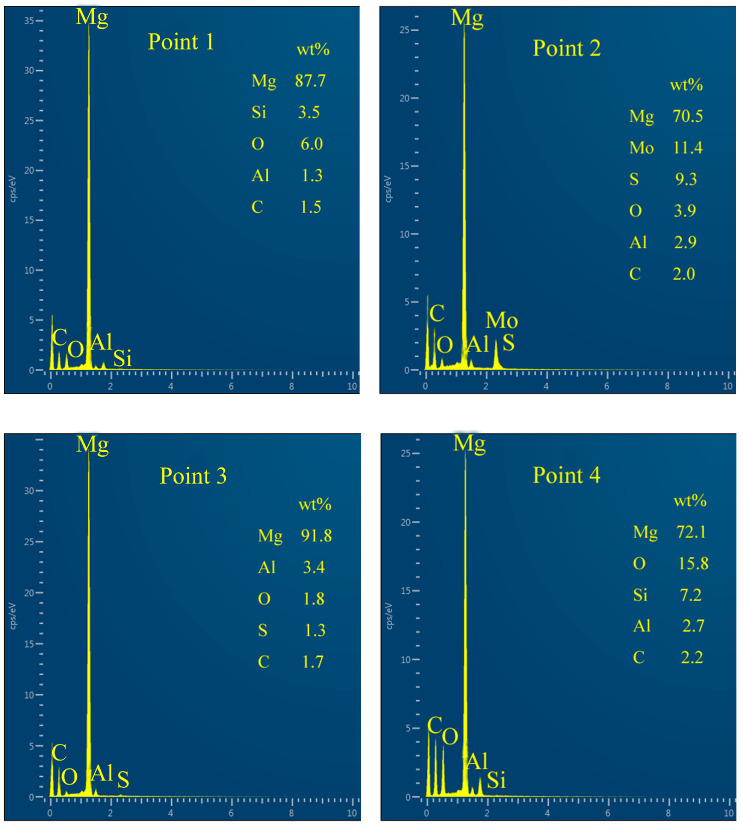
High-magnification FESEM-EDS spectra of the worn surfaces of the magnesium alloy lubricated by the SiO_2_:MoS_2_ (0.1:1.0) hybrid nanolubricants (**a**) and SiO_2_:MoS_2_ (0.5:1.0) hybrid nanolubricants (**b**) (3 N, 0.08 m/s, 30 min).

The above tribological results suggest that both friction reduction and wear resistance behaviors can be dramatically improved for the combinative addition of MoS_2_ nanoparticles and SiO_2_ nanoparticles into the base oil. In order to deeply understand the lubricant mechanism that is underlying the trends observed in the lubrication responses, the high magnification FESEM-EDS spectra of the worn surfaces lubricated by the SiO_2_:MoS_2_ (0.1:1.0) hybrid nanolubricants and SiO_2_:MoS_2_ (0.5:1.0) hybrid nanolubricants are chosen for detailed analyses in Figure 9. As shown in Figure 9a, it is clearly seen that the large particles deposit on the flat of the worn surfaces, while the granular particles fill up the grooves of the rubbing surfaces. Based on the EDS results, the large particles (point 1) are validated as MoS_2_, and the granular particles (point 2) are considered to be SiO_2_. It is suggested that the distribution of nanoparticles on the worn surface is substantially different on the microcosmic scale. The composition of the point 3 includes Mg, Al, O, S, and C. Combined with the XPS results, it is identified as MgSO_4_. As mentioned above, the nano-MoS_2_ protects the surface of the magnesium alloy in two ways. First of all, the MoS_2_ nanoparticles could be assembled on the rubbing surface due to the traction and compression formed by high contact stress. Intrinsically, the MoS_2_ shares the common structure of stiff, strongly bound planes held together by weak interlayer forces, which is what favors their sliding capability. The particular structure can provide low resistance for shear which thus results in friction reduction and wear resistance enhancement. Secondly, under the conditions of high pressure and high flash temperature caused by friction, the diffused MoS_2_ nanoparticles on the surface took tribo-chemical reaction with the rubbing surface to form molybdenum oxidized and magnesium sulfate, which had been demonstrated in the XPS analysis. The reaction mechanism can be directly compared with those acquired in the literature concerning the interactions of the MoS_2_ nanoparticles with the steel surface [41]. The reference described the possibilities as follows. One possibility is that the tribo-chemical film adhesion through the bonding of molybdenum with the oxygen was found in the oxide layer of the native steel surface. Therefore, a chemical reaction between molybdenum and the oxygen from MgO is chemically reasonable. The fact that the chemical reaction occurs between MgO and the MoS_2_ nanoparticles could be understood by a lower energy bonding between Mg and O than between Fe and O according to the Pearson hardness. Another possibility is that the oxide layer is quasi-immediate, wearing off at the early stage of the test, followed by S–Mg interactions and oxidization of the tribo-chemical film after its formation. The oxygen atoms could come from the air. Additionally, as in the past, it was proposed that water in the humid environment may prompt formation of MoO_3_ by direct oxidation. Giacomo Levita et al. [44] investigated the possibility of displacing a sulfur atom on the edge with the oxygen atom from the water molecule by ab initio calculations. The results showed that the MoS_2_ is more likely to absorb water along its edges instead of being oxidized by it at room temperatures. Even so, few experimental data have provided the evidence. To date, a complete understanding of the formation mechanism of the tribo-chemical film on the worn surface is still elusive. According to these results mentioned above, the adsorbtion of MoS_2_ nanoparticles, in conjunction with its reaction products with the magnesium alloy, forms a tribo-chemical film that dramatically improves the tribological behaviors.

In contrast, the friction reducing and anti-wear mechanism of spherical SiO_2_ nanoparticles is distinctly different from that of the lamellar MoS_2_ nanoparticles. Unlike nano-MoS_2_, nano-SiO_2_ is more likely to enter the contact area due to its infinitesimally tiny size and outstanding dispersion in the base oil, thereby resulting in faster running in stage (Figure 3).The existence of the spherical nano-SiO_2_ between the contact surfaces is often thought of as a wheel to help the easy sliding under normal load, although the rolling effect of nanoparticles as a lubricant additive is difficult to be directly observed during rubbing [45]. In this case, nanoparticles reduce the shear stress, thus resulting in friction reduction. Additionally, as reported by Mosleh Mohsen et al. [26], the filling of valleys of the contacting asperities will occur if the characteristic length *l* of flake-like nanoparticles is smaller than the peak-to-valley roughness of the harder surface which is equal to 4Ra, i.e., the condition of *l* < 4Ra applies. For the spherical particles, the diameter *d* of the particle should satisfy *d* < 0.67Ra. Therefore, the second mechanism of the significantly improved tribological performance of spherical SiO_2_ nanoparticles that are about 30 nm in diameter is ascribed to the fact that the nano-SiO_2_ spheres act as a third body material filling in the original pits and valleys on the surface of the magnesium alloy flat with 0.08 μm surface roughness, thus forming a thin physical film. The generated physical film is endowed with many crucial functions, including separating the contact surfaces to avert direct contact, enhancing the load-carrying capacity and decreasing the stress concentration. However, using nano-MoS_2_ platelets may be hard to fill in the microgaps because their length is larger than 4 Ra in the present study. Micro-cooperation occurred in succession between the shearing-sliding of the lamellar MoS_2_ nanoparticles and the filling of the spherical SiO_2_ nanoparticles during the sliding process. This observation is supported by relevant FESEM-EDS analysis in the present research (Figure 9a). If super abundant SiO_2_ nanoparticles were dispersed into the SiO_2_/MoS_2_ hybrid nanolubricants, SiO_2_ nanoparticles would tend to form irreversible agglomerates as shown in Figure 9b. The agglomerates limited the motion of nearby nanoparticles which generated a larger amount of stress concentrations leading to worse lubricating efficacy [46]. Additionally, the SiO_2_ nanoparticles possess excellent mechanical properties particularly with respect to the hardness (Hv = 1000 kgf/mm^2^); consequently, the excess hard SiO_2_ nanoparticles as an abrasive ploughs the soft surfaces of the magnesium alloy (Hv = 66.7 kgf/mm^2^) under the normal load. It will accelerate the abrasion of the magnesium alloy surface during the sliding process, thereby leading to the worse wear resistance. This was also verified by the inferior tribological properties of the nanolubricants with a high content of SiO_2_ nanoparticles, such as SiO_2_:MoS_2_ (0.2:1.0), SiO_2_:MoS_2_ (0.5:1.0), SiO_2_:MoS_2_ (0.7:1.0), and SiO_2_:MoS_2_ (1.0:1.0) in Figure 2 and Figure 4. Therefore, SiO_2_:MoS_2_ (0.1:1.0) hybrid nanolubricants exhibit the best tribological properties for synergetic lubrication even in the capacity of the carrying load and lubrication film stability tests. In view of the above-mentioned experimental results, a lubrication system based on the blends of SiO_2_ nanoparticles and MoS_2_ nanoparticles is established. The significant increase in the tribological performances by the SiO_2_/MoS_2_ hybrids as lubricant additives enables the sustainable and energy conserving lubricant system.

## 4. Conclusions

The tribological performances of nano-MoS_2_, nano-SiO_2_, and their hybrids as lubricant additives for magnesium alloy–steel pairs were evaluated using a reciprocating ball-on-flat tribotester. The conclusions can be summarized as follows from the experimental results.

(1) 1.0 wt % MoS_2_ nanolubricants, 0.1 wt % SiO_2_ nanolubricants, and SiO_2_/MoS_2_ hybrid nanolubricants presented superior friction-reducing and anti-wear behaviors as well as a high load-carrying capacity. The optimal friction reducing and anti-wear capability was obtained for the combinative addition of 1.0 wt % nano-MoS_2_ and 0.1 wt % nano-SiO_2_ into the base oil.
(2) 0.1 wt % SiO_2_ mixed with 1.0 wt % nano-MoS_2_ addition into the base oil showed a 21.8% reduction in the friction coefficient and 8.6% reduction in the wear volume compared with the 1.0 wt % MoS_2_ nanolubricants.
(3) Under the lubrication of the SiO_2_:MoS_2_ (0.1:1.0) hybrid nanolubricants, a complex tribo-chemical film that mainly contains MoO_3_ and MgSO_4_ through chemical reaction plus the absorbed nanomaterial which is from the lubricant additive itself is generated on the worn surfaces.

## Figures and Tables

**Table 1:** The primary characteristics of the EOT5# lubricant oil.

Primary Characteristics	Kinematic Viscosity (40 °C)	Density (15 °C)	Content of Saturates	Content of Aromatics	Contents of Sulfur
EOT5# lubricant oil	5.11 mm^2^/s	0.856 g/cm^3^	>90%	<1%	<0.03%
