# Correction: Xie et al. Synergistic Effect of MoS_2_ and SiO_2_ Nanoparticles as Lubricant Additives for Magnesium Alloy–Steel Contacts. *Nanomaterials* 2017, *7*, 154

**DOI:** 10.3390/nano12142364

**Published:** 2022-07-11

**Authors:** Hongmei Xie, Bin Jiang, Xingyu Hu, Cheng Peng, Hongli Guo, Fusheng Pan

**Affiliations:** 1 College of Mechanical and Electrical Engineering, Yangtze Normal University, Chongqing 408100, China; xiehongmei@yznu.cn (H.X.); guohongli1211@163.com (H.G.); 2 College of Materials Science and Engineering, National Engineering Research Center for Magnesium Alloys, Chongqing University, Chongqing 400044, China; fspan@cqu.edu.cn; 3 Chongqing Academy of Science and Technology, Chongqing 401123, China; 4 College of Materials Science and Engineering, Fudan University, Shanghai 200433, China; 16307110487@fudan.edu.cn

## Error in Figure

In the original publication [1], there was a mistake in Figure 7b,d as published. The authors have carefully checked the original files and found that it was an inadvertent mistake during the preparation of the manuscript. The corrected Figure 7 appears below.

## Text Correction

Additionally, we made the following changes in the text of the manuscript according to the corrected Figure 7d.

In Section 3.4, we replaced the following sentence:

“In marked contrast, the SiO_2_:MoS_2_ (0.1:1.0) hybrid nanolubricants produce even less surface damage with the smoothest worn surface and fewest grooves in a more random pattern.”

with the one below:

“In marked contrast, the SiO_2_:MoS_2_ (0.1:1.0) hybrid nanolubricants produce even less surface damage with wear debris and fewest grooves in a more random pattern.”

The authors apologize for any inconvenience caused and state that the scientific conclusions are unaffected. This correction was approved by the Academic Editor. The original publication has also been updated.

## Figures and Tables

**Figure 7:**
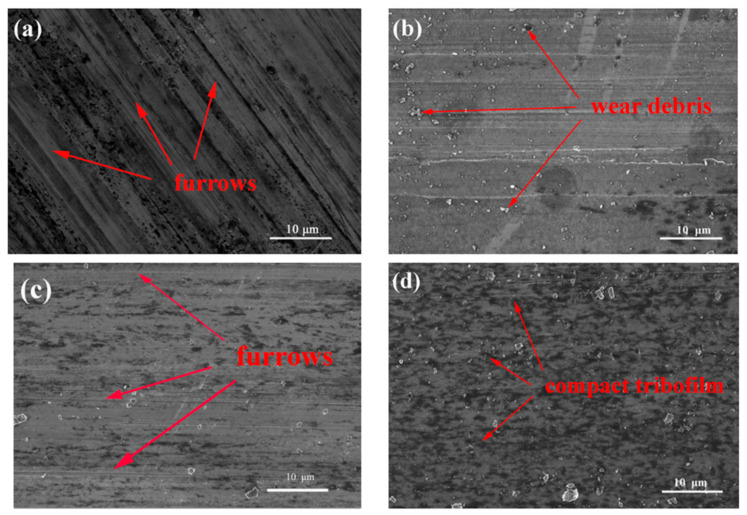
FESEM images of the worn surfaces on the magnesium alloy lubricated by: (**a**) base oil, (**b**) 0.1 wt % SiO_2_ nanolubricants, (**c**) 1.0 wt % MoS_2_ nanolubricants, and (**d**) SiO_2_:MoS_2_ (0.1:1.0) hybrid nanolubricants (3 N, 0.08 m/s, 0.5 h).
